# Interactions between Caveolin-1 (rs3807992) polymorphism and major dietary patterns on cardio-metabolic risk factors among obese and overweight women

**DOI:** 10.1186/s12902-021-00800-y

**Published:** 2021-07-01

**Authors:** Faezeh Abaj, Fariba Koohdani, Masoumeh Rafiee, Ehsan Alvandi, Mir Saeed Yekaninejad, Khadijeh Mirzaei

**Affiliations:** 1 Department of Community Nutrition, School of Nutritional Sciences and Dietetics, Tehran University of Medical Sciences (TUMS), Tehran, Iran; 2 Department of Cellular, Molecular Nutrition, School of Nutritional Sciences and Dietetics, Tehran University of Medical Sciences (TUMS), Tehran, Iran; 3 Department of Clinical Nutrition, School of Nutrition and Food Science, Isfahan University of Medical Sciences (IUMS), Isfahan, Iran; 4 School of Medicine, Western Sydney University, Campbelltown, NSW 2560, Australia; 5 Department of Epidemiology and Biostatistics, School of Public Health, Tehran University of Medical Sciences (TUMS), Tehran, Iran

**Keywords:** Diet, Gene-environment interaction, Caveolin-1, Cardio-metabolic, Personalized diet, Polymorphism

## Abstract

**Background:**

Caveolin-1 (CAV-1) is a cholesterol-dependent essential component located in caveolae. Several studies have been CAV-1 related to cardio-metabolic parameters in animal models, however, there are few studies in humans. Importantly, there is no study has investigated the interaction between *CAV-1 rs3807992* gene and dietary patterns (DPs) on cardio-metabolic risk factors.

**Methods:**

The current cross-sectional study was conducted on 404 overweight and obese women. Dietary intake was obtained from FFQ with 147 items. The CAV-1 genotype was measured by the PCR-RFLP method. The anthropometric measurements, serum lipid profile, and inflammatory markers were measured by standard protocols.

**Results:**

There was a significant interaction between *CAV-1 rs3807992* and healthy DP on high-density cholesterol (HDL) (P-interaction = 0.03), TC/HDL (P-interaction = 0.03) and high sensitivity C-reactive protein (hs-CRP) (P-interaction = 0.04); in A-allele carriers, higher following a healthy DP was related to a higher level of HDL and lower TC/HDL and hs-CRP. As well as, the significant interactions were observed between *CAV-1 rs3807992* and unhealthy DP in relation to triglyceride (TG) (P-interaction = 0.001), aspartate aminotransferase (AST) (P-interaction = 0.01) and monocyte chemoattractant protein-1(MCP-1) (P-interaction = 0.01); A-allele carriers were more following the unhealthy DP had lower levels of TG, AST and MCP-1.

**Conclusions:**

Our study revealed a significant gene-diet interaction between rs3807992 SNPs and DPs in relation to cardio-metabolic risk factors; A-allele carriers might be more sensitive to dietary composition compared to GG homozygotes. Following a healthy DP in A-allele-carriers may be improved their genetic association with cardio-metabolic risk factors.

## Background

The mortality rate of cardiovascular diseases (CVDs) rise to 23.6 million by 2030 [1]. Obesity-induced dyslipidemia, high blood pressure (BP) and inflammation have a key role in the pathogenesis of CVDs [2, 3]. Modifiable factors such as dietary intake and physical activity and non-modifiable including age, gender, and genes associated with the etiology of CVDs [4]. In several previous epidemiologic studies, the various nutrients separately have been considered without other components of dietary intakes [5, 6]. A complete analysis of dietary intakes can be provided precious insights into both nutrient intakes and dietary patterns (DPs) [7, 8]. Interestingly, DPs were associated with CVD risk in several studies [9–11]. Additionally, genome-wide association studies (GWAS) have discovered several usual variants correlated with CVD and obesity [12]. Caveolin-1 (CAV-1) is the most principal structural protein of caveolae, which is encoded by the genes CAV-1 [13]. Caveolae are found in most numerous cells but are especially abundant in adipocytes [14]. The physiological functions of caveolae are not fully illuminated. However, CAV-1 and caveolae are known to interact with a variety of physiologic and biologics pathways, including insulin sensitivity, lipid regulation especially cellular cholesterol and glucose homeostasis, maybe most importantly, cell signaling and receptors [15]. Some experimental studies have reported that CAV-1 plays a substantial role in the development of dyslipidemia, hypertension, and atherosclerosis [16–18]. Thus, has been reported CAV-1 single-nucleotide polymorphism (SNP) and the risk for various types of diseases and disorders [13, 19]. In some human studies, the two variant rs3807989 and rs926198 of CAV-1 genotypes may contribute to metabolic disorders and chronic diseases such as dyslipidemia, insulin resistance, metabolic syndrome, diabetes and CVDs [18, 20, 21]. Importantly, the association of *CAV-1 rs3807992* polymorphism with CVDs has not been reported up to now, present study is the first report to investigate this association.

On the other hand, animal studies have shown that various nutrients might modify the genetic susceptibility of CAV-1 to CVD or obesity risk [22, 23]. Particularly, CAV1 knockout mice have shown severe lipodystrophy and stay lean even after being fed high-fat diets [12]. In this regard, Cohen et al. [13] have found that rats fed with high-fat diet have higher CAV-1 expression. The previous animal studies on rabbits have revealed that, expression of the CAV-1 gene increase in response to high-cholesterol diet [24]. In another animal study, caveolin-1 has enhanced resveratrol-mediated cytotoxicity and transport in a hepatocellular carcinoma model [25]. In this regard, we have previously found insights about CAV-1 and insulin signaling in modifying dyslipidemia and fat composition in overweight and obese women [26]. Given the lack of human studies and contradictory results of animal and experimental studies, we conducted this study in order to determine the association between *CAV-1 rs3807992* and CVD risk factors and investigated whether dietary intake modulates this association.

## Methods

### Study population

The present study is a descriptive cross-sectional study carried out in Tehran, Iran. The recruitment of subjects was realized from the health center using a multi-stage cluster random sampling method. The inclusion criteria were women with age ≥ 18 years, health status, obesity and overweight (body mass index (BMI) ≥ 25 kg/m2). The participants were not pregnant, not the history of diseases including type 2 diabetes (T2D), CVDs, polycystic ovary syndrome (PCO), stroke, non-alcoholic fatty liver disease (NAFLD), Inflammatory disease include allergy, asthma, autoimmune diseases, coeliac disease and inflammatory bowel disease, hypertension, cancer, and thyroid, and also have not used weight loss program and supplements during our study. Additionally, women taking medications such as lipid-lowering (e.g. Atorvastatin, Cholestyramine, etc.), antihypertensive (Captopril, etc.) and blood glucose controlling (Metformin, etc.) and also their total calorie intake was not in range between 800 and 4200 were excluded. A self-administered questionnaire was provided from all participants for their health status and the exclusion criteria of the study. Finally, total 404 eligible women were included into the present study. All protocols of this study were conducted in accordance with Helsinki Declaration and approved by the Ethical Committee of the Tehran University of Medical Sciences (TUMS) (NO: 97–03–161-41,017). All of the participants completed a written informed consent form before taking part in the study.

### Dietary assessments

Dietary intakes were assessed by expert dietitians using a validated 147-items semi-quantitative food frequency questionnaire (FFQ) (Table 1) [27]. Subjects were reported the frequency of each food item consumed on a daily, weekly, monthly, or yearly during the past year. Then, was converted to grams per day using household measures. Total energy and dietary nutrients were assessed by the Iranian Food Composition Table (FCT) and N4 software. Finally, 17 food groups were extracted for an examination of DPs of dietary intake.

**Table 1:** Food Frequency Questionnaire (FFQ)

No	Food	code	Times per weak				Times per months			Times per year		
			6 +	4–5	2–3	1	5–6	2–4	1	1	Never or Less than once	Note
1	White bread (Lavash bread)											
2	White bread (Barbari bread)											
3	Wholemeal bread (Sangac bread)											
4	White bread (Taftun bread)											
5	White bread (baguette and other fantasy bread)											
6	Cooked white rice											
P	Macaroni											
8	Potatoes											
9	Fried potatoes											
10	Noodles (ash) boiled											
11	Cake and Biscuit											
12	Corn											
13	Barely or Buckwheat											
14	Lentile											
15	Bean											
16	Pea											
17	Chickpea											
18	Soya											
19	Split Pea											
20	Meat											
21	Mincemeat											
22	Chicken, hens (breast)											
23	Fish											
24	Canned Tuna fish											
25	Sausage and Martadela											
26	Egg											
27	Pizza											
28	Milk Cocoa											
29	Low-fat milk											
30	Full-fat milk											
31	Yogurt											
32	Cheese											
33	*Yogurt drink (Dough)*											
34	Cream milk and Cream											
35	Ice cream											
36	Butter											
37	Kashk (A condensed and salted form of yogurt)											
38	Lettuce											
39	Tomato											
40	Cucumber											
41	Culinary vegetables											
42	Cooked vegetable											
43	Pumpkin											
44	Courgette											
45	Cooked eggplant											
46	Celery											
47	Pea											
48	Green beans											
49	Carrot											
50	Garlic											
51	Onion											
52	All type of Cabbage											
53	All type of pepper											
54	Cooked spinach											
55	Turnip											
56	Tomato ketchup/Tomato puree											
57	Pickle											
58	Cucumber pickles											
59	Cantaloupe and Honey dew melon											
60	Melon											
61	Watermelon											
62	Pear											
63	Apricot											
64	Cherry / Black cherry											
65	Apple											
66	Peach											
67	Nectarine											
68	Plum Green											
69	Fig											
70	Grape											
71	Kiwi											
72	Orange											
73	Persimmon											
74	Tangerine											
75	Pomegranate											
76	Date											
77	Prune (Yellow and Red)											
78	Strawberry											
79	Banana											
80	Lemon											
81	Lime											
82	Natural fruit juice											
83	Raisins											
84	Berriy											
85	Dried fruits											
86	Green olive											
87	Canned fruits											
88	Industrial l fruit juice											
89	Solid vegetable’s oil											
90	Animal’s Oil											
91	Olive oil											
92	Liquid oil											
93	Mayonnaise sauce											
94	Almond											
95	Peanut											
96	Walnut											
97	Pistachio											
98	Hazelnut											
99	Seed											
100	Cube suger or Noghl											
101	Suger											
102	Honey											
103	Jam											
104	Industrial beverage											
105	Sweet											
106	Gaz											
107	Sohan											
108	Chocolate											
109	Tea											
110	Salt											
111	Coffee											
112	Limejuice											
113	Candy/Sugerpalm											
114	Mushroom											
115	Homemade halva											
116	Sugared halva											
117	All type of spices											

### General and anthropometric assessments

General data such as age, educational level, marital status, family history of obesity was collected via standard questionnaires. Body weight and height were measured using a digital scale (Seca, Germany) and tape measure with a precision of 100 g and 0.1 cm, respectively, when the subjects were minimally clothed and not wearing shoes in a standing position. Waist circumference (WC, cm) was measured at the narrowest part of the abdomen to the nearest 0.1 cm. BMI was calculated as weight (kilograms) divided by the height (meters) squared. Physical activity was assessed by the validated International Physical Activity Questionnaire [28].

### Laboratory tests

All samples were collected after overnight fasting (10–12 h) at the Nutrition and Biochemistry laboratory of the School of Nutritional Sciences and Dietetics, TUMS. indices of serum lipids and lipoproteins (total cholesterol (TC), HDL-C, low-density lipoprotein cholesterol (LDL-C) and TG), liver enzymes (alanine aminotransferase (ALT) and aspartate aminotransferase (AST)), hs-CRP, MCP-1, interleukin 1 beta (IL-1β) and transforming growth factor beta1(TGF-β1) were measured via standard protocols. Plasminogen activator inhibitor-1 (PAI-1) (Human PAI-1*96 T ELISA kit Crystal Company) was measured in triplicate.

### DNA analysis

Genomic DNA was extracted from the whole blood sample by a Mini Columns kit (Type G; Genall; Exgene) based on the manufacturer’s guidebook. The *CAV-1 rs3807992* SNP (major allele: G; minor allele: A) was genotyped by PCR-RFLP (polymerase chain reaction-restriction fragment length polymorphism) technique as follows. The PCR amplification of the genomic DNA fragment for rs3807992 was performed by the forward primer 3′ AGTATTGACCTGATTTGCCATG 5′ and reverse primer 5′ GTCTTCTGGAAAAAGCACATGA 3′. The final volume of PCR product was contained 20 μl including 1 μl extracted DNA, 1 μl forward primers, 1 μl reverse primers, 7 μl distilled water, and 10 μl Taq DNA Polymerase Master Mix (Amplicon; Germany). PCR cycles were designed with initial denaturation step at 94 °C for 3 min (40 cycles), annealing at 53 °C for 30 s and 30 s of extension at 72 °C, eventually a final extension at 72 °C for 3 min. Amplified DNA (10 μl) was digested using 0.5 μl of Hin1II (NlaIII) restriction enzyme (Fermentase, Germany) at 37 °C overnight. Finally, electrophoresis of the PCR products was performed on 3.5% agarose gel. Three DNA fragments appeared with different lengths: homozygous AA (1 band: 213 bp), heterozygous GA (3 bands: 118 & 95 & 213 bp and homozygous GG (2 bands: 118 & 95 bp). Importantly, 10% of the samples were directly sequenced for confirmation of the PCR-RFLP results. The sequencing process performed using the ABI PRISM 3730 automated sequencer (Applied Biosystems, Foster City, Calif, USA).

### Statistical analyses

The normality distribution was checked by the Kolmogorov- Smirnov test. Non-normal data including TG and AST (*p* < 0.05) were normalized by logarithmic transformation. Continuous variables were expressed as mean ± SD. Pearson’s chi-square test was used for the Hardy-Weinberg Equilibrium (HEW). Genotype groups were considered as a dominant inherent model. DPs were examined using principal factor analysis (PCA) and factors were extracted by varimax rotation to ensure unrelated conditions and improve interpretability. Eventually, factors were obtained by considering an eigenvalue of more than 1.5 and a scree plot. The factor scores were evaluated as the sum of each factor loading ≥|0.3| and the reference daily intake of each food correlated to the DPs. These factors containing: vegetables, low-fat dairy, starchy vegetables, fruits, legumes, fish, nuts& olive, red meat, poultry, spices, sweet snack & drinks, processed foods, high-fat dairy and Tea& coffee. Thus, the high score was associated with more adaptation to the extracted pattern dietary. Subjects were categorized into tertiles (T1-T3) by scores of 17 factors for further analyses. Differences of continuous variables in genotype groups and DPs were compared by the independent-sample t-test and the One-Way ANOVA test in the crude model, respectively. ANCOVA analysis was performed for adjustment model (adjusting for age, physical activity and energy intake). A generalized linear model (GLM) was exerted to analyze the interactions between CAV-1 polymorphism (rs3807992) and DPs concerning metabolic risk factors of cardiovascular disease. Data were analyzed using Statistical Package for Social Sciences (SPSS Inc., Chicago, IL, version 25) and *P*-value < 0.05 was considered as significant.

## Results

### Study population characteristics

The present study was carried out on 404 women with a mean (SD) age of 36.67 ± 9.1 years. The majority of women were married (70.8%) and had a university education (54%) and a history of obesity (71.2%). The genotypic frequency among participants was as follow: GG (50%), AG (23.3%) and AA (26.7%). Further, the minor frequency allele (A) was 38% in this study. The genotype distributions had a deviation from in Hardy-Weinberg equilibrium (*P* < 0.05).

### Difference in means of cardio metabolic variables between *CAV-1 rs3807992* genotypes

The comparison of variables including age, BMI and biochemical parameters according to two genotypes groups (GG and AG + AA) is given in Table 2. Results show that A-allele carriers have significantly higher and lower BMI and age, respectively, compared to GG homozygotes (*P* = 0.02 and *P* = 0.05, respectively). Additionally, there was a significant association between CAV-1 polymorphism (rs3807992) and serum LDL-C, HDL in both unadjusted (crude) and adjusted models (adjusted for age, physical activity and energy intake) (*P* < 0.0001 and *P* = 0.006, *P* = 0.003 and *P* = 0.001, respectively), TC/HDL in unadjusted model (*P* = 0.01) and TC in the adjusted model (*P* = 0.04). In particular, serum LDL-C, HDL-C, and TC concentration were significantly higher in GG homozygous compared to A-allele carriers. Although, the TC/HDL ratio was significantly lower in those. Moreover, there was no significant association between this polymorphism and other biochemical parameters including TG, inflammatory markers, and liver enzyme in both crude and adjusted models (*P* > 0.05) (Table 2).

**Table 2:** The association between metabolic markers and the genotypes of Cav-1 rs3807992 polymorphism

	CAV-1 (rs3807992)		*P* value*	*P*-value**
	GG	(AG + AA)		
**Age(year)**	37.56 ± 9.49	35.75 ± 8.78	0.05	
**BMI**	30.68 ± 4.01	31.66 ± 4.46	**0.02**	
**TC(mg/dl)**	186.76 ± 33.74	182.71 ± 37.36	0.30	**0.04**
**HDL-C(mg/dl)**	49.07 ± 11.16	44.04 ± 10.16	**< 0.0001**	**0.003**
**LDL-C(mg/dl)**	98.80 ± 22.66	91.27 ± 25.07	**0.006**	**0.001**
**LDL.HDL**	2.07 ± 0.53	2.14 ± 0.64	0.58	0.56
**TC.HDL**	3.93 ± 0.89	4.43 ± 1.91	**0.01**	0.23
**TG(mg/dl)**	113.11 ± 51.20	133.31 ± 84.14	0.13	0.14
**ALT (IU.L)**	18.27 ± 7.44	17.95 ± 8.29	0.41	0.24
**AST(IU.L)**	19.04 ± 13.95	20.14 ± 14.19	0.61	0.88
**hs-CRP(mg/L)**	4.18 ± 4.40	4.33 ± 4.76	0.94	0.68
**MCP-1(ng/ml)**	51.49 ± 102.26	48.42 ± 81.14	0.8	0.99
**PAI.1(ng/ml)**	19.24 ± 38.43	12.70 ± 17.42	0.16	0.94
**IL1B(pg/ml)**	0.36 ± 0.54	0.37 ± 0.53	0.94	0.95

*SD* Standard deviation; *BMI* Body mass index; *TC* Total cholesterol; *HDL* High density lipoprotein; *LDL* Low density lipoprotein; *TG* Triglyceride; *ALT* Alanine aminotransferase; *AST* Aspartate aminotransferase; *hs-CRP* High-sensitivity C-reactive protein; *MCP-1* monocyte chemoattractant protein; *PAI-1* Plasminogen Activator Inhibitor 1; *IL1B* Interleukin 1 beta

* *P*-value for crude model

**P value for adjusted model (BMI considered as collinear and this variable adjusted for Age, physical activity level, energy intake)

### Difference in means of cardio metabolic variables between major DPs

The factor-loading scores for each DP are represented in Table 3. Two major dietary patterns were identified; the healthy dietary pattern was loaded extremely on raw vegetables, fruits, starchy vegetables, legumes, low-fat dairy, nuts and olives, red meat, poultry, fish, and spices, whereas the unhealthy dietary pattern comprised sweet snack& drinks, refined grain, salty snack, processed foods, animal oil, high-fat dairy, coffee &tea, and with low for legumes.

**Table 3:** Factor loadings for three identified food patterns

Food groups	Food patterns	
	Healthy dietary pattern	Unhealthy dietary pattern
Vegetables	0.66	
Low-fat-dairy	0.570	
Starchy vegetables	0.564	
Fruits	0.534	
Legumes	0.479	−0.381
Fish	0.447	
Nuts& Olive	0.437	
Red-meat	0.435	
poultry	0.434	
Spices	0.368	
Sweet snack & drinks		0.633
Refined grains		0.478
Salty snack		0.476
Processed foods		0.456
Animal oil		0.422
High-fat-dairy		0.373
Tea& Coffee		0.305
Variance(%)	14.91	7.69
Total variance (%) = 22.61		

Values are factor loadings of food patterns measured by

factor analysis (*n* = 404). Factor loadings below ±0.3 are not shown. Eigenvalues =1.5

The results displayed that women in the highest tertile (T3) of the unhealthy pattern were significantly younger than those in T1 and T2 (*p* = 0.002). Women in T1 of the healthy pattern had significantly higher serum TC concentration than those in T2 and T3 of the same pattern in the unadjusted model (*p* = 0.01). Although, this significance was less in the adjusted model (for age, energy intake, physical activity and BMI) (*P* = 0.05) (Table 4**)**.

**Table 4:** Characteristics of study population based on tertile of dominant dietary patterns intake

Healthy dietary pattern						Unhealthy dietary pattern				
	T_**1**_	T_**2**_	T_**3**_	Pvalue*	Pvalue**	T_**1**_	T_**2**_	T_**3**_	*P* value*	*P*-value**
**Age(year)**	37.73 ± 9.054	36.27 ± 9.541	36.22 ± 8.94	0.32		38.45 ± 8.67	37.32 ± 9.42	34.48 ± 9.06	**0.002**	
**BMI(kg/m**^**2)**^	30.77 ± 3.88	30.58 ± 3.67	31.56 ± 4.03	0.1		31.09 ± 4.06	31.08 ± 3.85	31.69 ± 4.92	0.42	
**SBP(mmHg)**	111.64 ± 12.75	110.34 ± 17.38	112.07 ± 14.19	0.7	0.83	112.75 ± 13.85	107.6 ± 16.59	113.03 ± 14.18	**0.02**	0.09
**DBP(mmHg)**	78.72 ± 8.47	77.04 ± 12.49	76.92 ± 9.81	0.44	0.42	78.24 ± 8.71	75.11 ± 12.18	78.78 ± 10.4	**0.04**	**0.03**
**TC(mg/dl)**	191.32 ± 36.72	176.15 ± 37.16	187.44 ± 33.61	**0.01**	0.05	185.39 ± 33.75	182.59 ± 40.26	186.49 ± 35.44	0.7	0.82
**HDL-C(mg/dl)**	47.70 ± 11.01	45.63 ± 9.24	47.03 ± 12.02	0.66	0.52	46.82 ± 11.32	47.25 ± 11.19	46.28 ± 10.07	0.93	0.44
**LDL-C(mg/dl)**	96.85 ± 26.38	91.08 ± 23.28	96.63 ± 22.78	0.3	0.05	97.32 ± 23.90	91.28 ± 24.53	95.15 ± 23.95	0.19	0.7
**LDL/HDL**	2.10 ± 0.65	2.05 ± 0.53	2.14 ± 0.59	0.6	0.07	2.15 ± 0.61	1.98 ± 0.52	2.13 ± 0.61	0.15	0.59
**TC/HDL**	4.30 ± 2.04	3.97 ± 1.02	4.22 ± 1.37	0.39	0.13	4.14 ± 1.19	4.13 ± 2.05	4.22 ± 1.28	0.66	0.49
**TG(mg/dl)**	109.08 ± 54.2	127.01 ± 69.5	128.52 ± 81.22	0.13	0.25	131.56 ± 85.09	113.97 ± 57.53	118.03 ± 59.76	0.35	0.39
**ALT (IU.L)**	17.24 ± 5.77	17.13 ± 5.68	18.96 ± 9.48	0.39	0.43	18.86 ± 8.35	16.83 ± 5.30	17.50 ± 7.62	0.21	0.08
**AST(IU.L)**	18.30 ± 9.86	17.70 ± 10.24	21.16 ± 16.77	0.31	0.73	20.38 ± 14.65	17.44 ± 10.22	19.26 ± 13.12	0.19	0.18
**MCP-1 (ng/ml)**	40.14 ± 85.61	55.33 ± 91.90	56.47 ± 99.42	0.49	0.66	59.92 ± 117.5	52.76 ± 69.74	37.61 ± 73.5	0.32	0.61
**hs-CRP(mg/L)**	4.44 ± 5.05	4.19 ± 4.82	4.06 ± 4.12	0.91	0.26	4.27 ± 4.72	4.09 ± 4.78	4.28 ± 4.41	0.53	0.8
**PAI.1(ng/ml)**	9.21 ± 12.33	14 ± 26.21	24.03 ± 40.49	0.1	0.13	18.9 ± 39.07	16.57 ± 23.74	11.46 ± 18.37	0.43	0.39

Values are presented as Mean ± SD. *BMI* Body mass index; *TC* Total cholesterol; *HDL* High density lipoprotein; *LDL* Low density lipoprotein; *TG* Triglyceride; *ALT* Alanine aminotransferase; *AST* Aspartate aminotransferase; hs-*CRP* High-sensitivity C-reactive protein; *MCP-1* monocyte chemoattractant protein; *PAI-1* Plasminogen Activator Inhibitor 1

* *P*-value for crude model **P value for adjusted model (BMI considered as collinear and this variable adjusted for Age, physical activity level, energy intake)

### Interaction between major DPs and *CAV-1 rs3807992* genotypes on cardio-metabolic risk factors

**Fig. 1:**
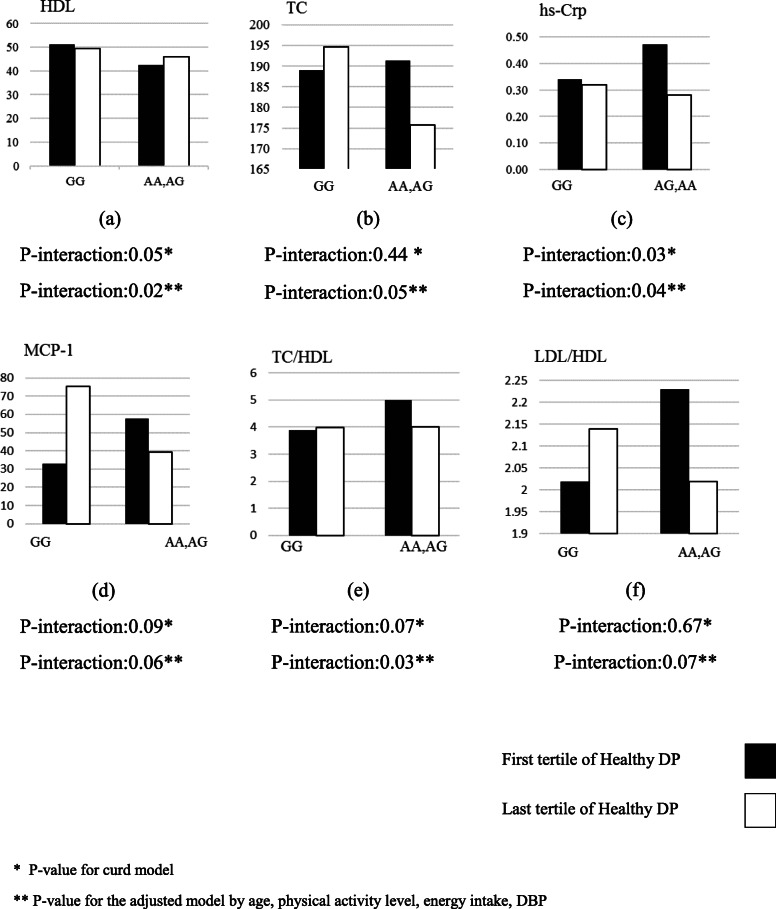
The interaction between Cav-1 SNP rs807992 and healthy dietary pattern on; (**a**) HDL, (**b**) TC, (**c**) hs-Crp, (**d**) MCP-1, (**e**) TC/HDL (f) LDL/HDL

**Fig. 2:**
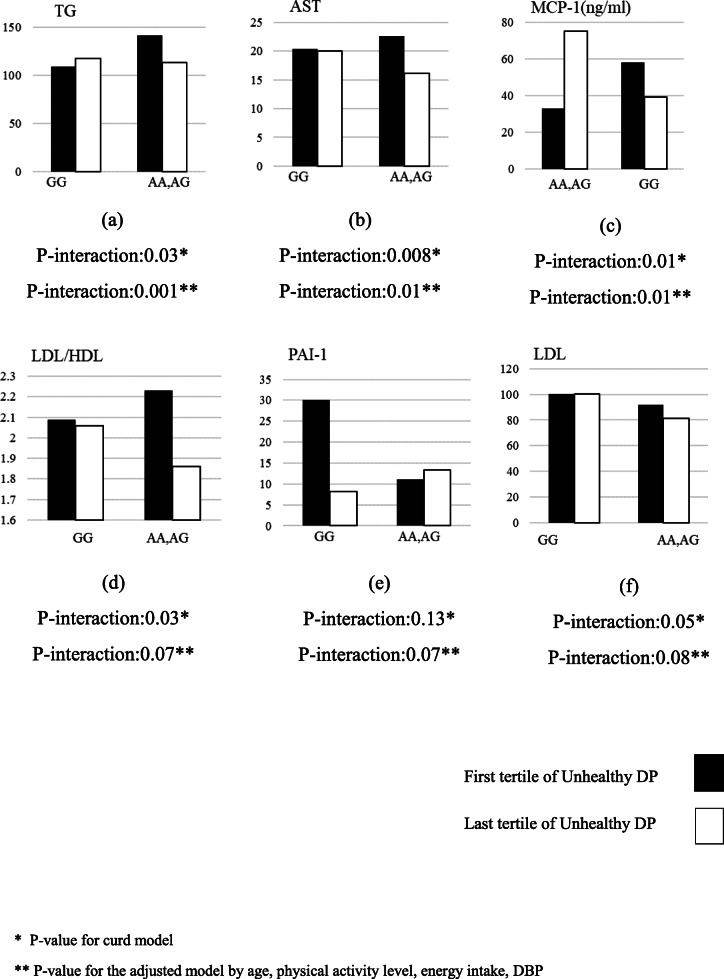
The interaction between Cav-1 SNP rs807992 and Unhealthy dietary pattern on; (**a**) TG, (**b**) AST, (**c**) MCP-1, (**d**) LDL/HDL, (**e**) PAI-1and (**f**) LDL

The interaction effects between CAV-1 variants at rs3807992 and two DPs (healthy and unhealthy) on cardio-metabolic markers including TC, HDL, LDL, TC, TC/HDL, LDL/HDL, TG, ALT, AST, hs-CRP, MCP-1, PAI-1 and IL-1β were shown in Tables 5 and 6. Furthermore, the significant interactions were reported via bar chart in Figs. 1 and 2.

**Table 5:** Interactions between CAV-1 rs3807992 and healthy dietary intake on CVDs risk factors

	β (95%CI) (AA + AG) 1(Ref) GG			
	***Crude***	P*	***Adjusted***	P**
TC (mg/dl)	−0.02 (−.07,0.03)	0.44	− 0.04 (−.09,0.006)	0.08
HDL-C (mg/dl)	6.67 (−.13,13.48)	0.05	7.74 (−0.76,14.72)	**0.03**
LDL-C (mg/dl)	7.68 (−7.43,22.81)	0.31	−2.53(−18.19,13.12)	0.75
TC/HDL	−0.86 (−1.81,0.08)	0.07	−1.01 (− 1.97,0.05)	**0.03**
LDL/HDL	−0.07 (−.44,0.29)	0.67	− 0.31 (−.67,0.04)	0.08
TG (mg/dl)	−0.09 (− 0.61,0.42)	0.72	−4.33 (−47.04,38.37)	0.84
ALT (IU.L)	−2.64(−11.06,5.7)	0.53	−3.38 (− 12.27,5.5)	0.45
AST (IU.L)	−0.95(−5.55,3.97)	0.74	−1.34 (−6.29,3.61)	0.59
hs-CRP (mg/L)	−0.38 (− 0.74, − 0.02)	**0.03**	−0.34 (− 0.68,-0.007)	**0.04**
MCP-1 (ng/ml)	−51.88 (− 112.93,9.17)	0.09	−60.89 (− 124.51,2.72)	0.06
PAI-1 (ng/ml)	−12.45 (− 34.91,10.01)	0.27	− 12.42(− 36.24,11.4)	0.30
IL1B (pg/ml)	− 0.16 (− 0.51,0.18)	0.35	− 0.33 (− 0.69,0.02)	0.06

Values are represented as β (95%CI)

A significant *p*-values are indicated in bold (significance considered *p* < 0.05)

GLM was performed to identify significant differences between tertiles of healthy DP and CAV-1 rs3807992

P* = with unadjusted (crude) model

P** = with adjustments for potential confounding factors including (age, energy intake, physical activity, DBP)

**Table 6:** Interactions between CAV-1 rs3807992 and unhealthy dietary intake on CVDs risk factors

	β (95%CI) (AA + AG) 1(Ref) GG			
	***Crude***	P*	***Adjusted***	P**
TC (mg/dl)	6.30 (−15.08,27.96)	0.56	9.90 (− 12.33,32.14)	0.38
HDL-C (mg/dl)	3.66(− 2.78,10.11)	0.26	5.27(−1.64,12.19)	0.13
LDL (mg/dl)	−14.08 (− 28.70,0.52)	0.05	− 0.061 (−.131,0.009)	0.08
TC/HDL	−0.38(− 1.32,0.55)	0.41	− 0.29(− 1.33,0.74)	0.57
LDL/HDL	− 0.39 (− 0.75,-0.03)	**0**.**03**	−0.33 (− 0.69,0.02)	0.07
TG (mg/dl)	−70.76 (113.80,27.72)	**0.001**	−64.63 (103.68,25.58)	**0.001**
ALT(IU.L)	−2.6 (−7.26,2.05)	0.27	−8.01(−16.92,0.89)	0.07
AST (IU.L)	−0.19 (− 0.33,-0.05)	**0.008**	− 0.18 (− 0.33,0.04)	**0.01**
hs-CRP (mg/L)	−1.08(− 3.93,1.76)	0.45	− 1.59(− 4.79,1.59)	0.32
MCP-1(ng/ml)	73.43 (13.01,133.859)	**0.01**	82.27 (17.42,147.129)	**0.01**
PAI-1(ng/ml)	17.12 (−5.52,39.78)	0.13	20.96 (−2.21,44.13)	0.07
IL1B (pg/ml)	0.09 (−0.27,0.46)	0.61	−0.25 (− 0.6,0.09)	0.15

Values are represented as β (95%CI)

A significant p-values are indicated in bold (significance considered *p* < 0.05)

GLM was performed to identify significant differences between tertiles of unhealthy DP and CAV-1 rs3807992

P* = with unadjusted (crude) model

P** = with adjustments for potential confounding factors including (Age, energy intake, physical activity, DBP)

There is a gen-diet interaction for healthy pattern and CAV-1 polymorphism (rs3807992) on HDL (β: 7.74, 95%CI: − 0.76 to 14.72, P:0.03), TC/HDL (β: -1.01, 95%CI: − 1.97 to 0.05, P:0.03), and hs-CRP (β: -0.34, 95%CI: − 0.68 to − 0.007, P:0.04), in adjusted model (adjusting for age, energy intake, physical activity and DBP) (Table 5). Thus, the A-allele carriers who were placed in the last tertile of the healthy DP had a higher HDL-C level and lower TC/HDL and hs-CRP compared to GG homozygotes (Fig. 1). No significant interactions were found between ***CAV-1 rs3807992*** variants and healthy dietary DP intake for other metabolic-related traits (*p* > 0.05).

Furthermore, there is significant interactions between unhealthy DP and rs3807992 on TG (β: -64.63, 95%CI: 103.68 to 25.58, P:0.001), AST (β: -0.18, 95%CI: − 0.33 to 0.04, P:0.01) and MCP-1 (β: 82.27, 95%CI: 17.42 to 147.12, P:0.03) in adjustment model (adjusting for age, energy intake, physical activity and DBP) (Table 6). In particular, A-allele carriers were characterized by lower serum TG and AST when had the highest following an unhealthy DP compared to GG homozygote. (Fig. 2). Also, risk-allele carriers who consumed higher unhealthy DP had higher inflammatory marker include MCP-1 concentration. No significant interactions were found between ***CAV-1 rs3807992*** variants and unhealthy dietary DP intake for other metabolic-related traits (p > 0.05).

## Discussion

The present study provides information on the interaction between dietary patterns and genetic polymorphism of CAV-1 (rs3807992) in association with cardio-metabolic traits. Our findings represent that following a healthy DP was associated with lower serum TC level. Besides, in our study younger women have the highest unhealthy DP score. In particular, following an unhealthy DP was associated with higher BP. Additionally, findings showed that A-allele carriers of the rs3807992 polymorphism had higher BMI and lower TC, HDL-C, and LDL-C compared with GG homozygous. In the current study, we observed A-allele carriers of rs3807992 had a higher serum HDL-C, lower TC/HDL and hs-CRP concentration when placed in the last tertile of healthy DP compared to GG homozygotes. Another novel significant interaction was found between rs3807992 polymorphism and unhealthy DP on TG, AST, and MCP-1. In particular, A-allele carriers with the highest following an unhealthy DP had lower HDL, AST, and MCP-1 concentrations compared to the GG genotype. It is now well accepted, that the prevalence of obesity and CVD is due to changes in DPs [29, 30]. In line with our study, several studies have shown the protective effects of healthy DPs as an independent factor on decrease of CVD risk, which has not been reported precise mechanisms yet. However, a possible mechanism has suggested that high content of phytosterols, pectin and beta-glucagon in healthy patterns can decrease intestinal absorption of cholesterol [31, 32]. Moreover, in line with our study, several studies have shown that subjects who follow the unhealthy DP (comprise of foods with high-sodium and low potassium and magnesium and also caffeine) have statistically significant higher DBP [33, 34]. It can be due to raises renin activity disruption and systemic vascular resistance [35–37].

Although the underlying mechanism of the CAV-1 gene on cardio-metabolic risk factors is not completely known, some studies have shown that CAV-1 regulated the intracellular transport of fatty acids and cholesterol through direct binding to them [38–40]. Given this mentioned above, CAV-1 plays an important role in lipid and lipoprotein metabolism such as HDL-C, TC, VLDL and TG [17, 41–43]..

Moreover, our study revealed that risk-allele carriers who follow a healthy DP have higher HDL and lower hs-CRP and MCP-1 levels. It has now been suggested that diet and plasma-derived nutrients may modulate metabolic biomarkers through interacting with caveolae-associated cellular signalling [44]. In this regard, we revealed higher polyunsaturated fatty acid consumption might attenuate the *CAV-1 rs3807992* associations with metabolic syndrome (MetS), and risk-allele appeared to have a higher risk of MetS, associated with higher saturated fatty acid consumption [45]. In regarding to the interaction between CAV-1 variant and diet on inflammatory markers, some studies have suggested that CAV-1 binds to endothelial nitric oxide synthase (eNOS) and HDL receptor in the caveolae and inhibits their activity, but the exact mechanism is not clear yet. Thus, the anti-inflammatory diet can be displaced the CAV-1 from caveolae to the cytoplasm which led to a decrease in the CAV-1 level, as a result, disappears the inhibitory effects on HDL and eNOS receptors [31]. As respects, Oberleithner et al. have claimed that serum sodium and potassium can regulate the binding of eNOS to the caveolae membrane and its activity [32]. Hence, the favourable effects of the healthy DP may be attributed to components such as vegetables and fruits, which more influence on balance potassium and sodium. Besides, this study revealed that A-allele carriers who had more follow unhealthy DP have lower levels of serum TG and LDL. These findings are advocated by Philippe et al., in which CAV-1(+/+) mice fed to high cholesterol has lower TC and TG levels compared to CAV-1(−/−) [33]. According to our finding, probably the lower AST and TG concentration in A-allele carriers following an unhealthy DP be dependent on the caffeine intake. According to probable mechanism, the low AST and TG concentration in A-allele carriers following an unhealthy DP be dependent on the caffeine intake. Regarding, there is particular region of a candidate gene for caffeine intake near to the CAV-1 gene [59], we have suggested a possible interaction between caffeine and CAV-1, and have hoped that will prove completely in further research. Besides, reduced NO production due to increased CAV-1 expression has been thought to result in prolonged exposure to high glucose, which may play a potential role in inflammatory pathways and development of inflammation [34]. Hence, it is not surprising that higher following an unhealthy DP can increase MCP-1 levels by altering the expression of CAV-1 and other genes. However, because of limitations in the financial source, we could not perform western blot analysis to find out whether rs-3,807,992 SNP alters the expression of CAV-1.

### Limitation and strength

Limitations of the present study including the cross-sectional design, so any causality cannot be argued; the use of FFQ for dietary assessing, which may have resulted in memory bias; small sample size, which may have led to weak statistical to determine significant results and we could not eliminate all confounder factors, which can be affected on our results. Furthermore, our participants were from the Iranian country which may not be generalized due to racial and regional differences (52). Finally, we did not include a normal-weight participant due to financial constraints and instead focused on overweight and obese women as high-risk categories for metabolic traits [61]. However, as this is the first study of its kind, we propose that future studies focus on women of normal weight. Despite the limitations mentioned above, this is the first effort to study the interaction between CAV-1 rs3807992 polymorphism and DPs on cardio-metabolic risk factors. Recognition of these gene-diet interactions could be determining in prescribe personalized nutritional recommendations for the improvement and management of CVD risk. Finally, these results can be used in combination with a patient’s genetic history to provide more applicable and tailored nutritional advice for preventing or attenuating cardiovascular disease in overweight and obese women.

## Conclusions

In conclusion, our results were in agreement with the differential-susceptibility model, which is defined as the risk alleles that could be modified with environmental factors by positive or negative effects. In particular, A-allele carriers might be more sensitive to dietary composition compared to GG homozygotes. Our study revealed a significant gene-diet interaction between rs3807992 SNPs and DPs in relation to cardio-metabolic risk factors; following a healthy DP in A-allele-carriers may be improved their genetic association with HDL, TC/HDL, and hs-CRP, as well as following an unhealthy DPs may be modified on genetic susceptibility to TG, AST, and MCP-1. We believe that our research will consider as a framework for future studies on CAV-1 gene and diet interaction. Large prospective studies are needed to confirm the present results.

## Data Availability

The data are not publicly available due to containing private information of participants. Data are however available from the authors upon reasonable request and with permission of Khadijeh Mirzaei.

## Abbreviations

AST: Aspartate aminotransferase
ApoA-I: Apolipoprotein A-I
BIA: Bioelectrical impedance analysis
BMI: Body mass index
bp: Base pair
CVD: Cardiovascular disease
DBP: Diastolic blood pressure
DNA: Deoxyribonucleic acid
DP: Dietary pattern
eNOS: Endothelial nitric oxide synthase
FFQ: Food frequency questionnaire
GLM: General linear model
GWAS: Genome-wide association studies
HDL: High-density lipoprotein cholesterol
IR: Insulin receptor
hs-CRP: High sensitivity C-reactive protein
IPAQ: International physical activity questionnaire
LDL: Low-density lipoprotein
MA: Minor allele
PCR: Polymerase chain reaction
RFLP: Restriction fragment length polymorphism
SNP: Single nucleotide polymorphism
TC: Total cholesterol
TG: Triglyceride
VAT: Visceral adipose tissue
WC: Waist circumference
WHO: World health organization
